# Social services for the elderly: a multivariate perspective study

**DOI:** 10.3389/fpsyg.2023.1297349

**Published:** 2023-11-24

**Authors:** María-Concepción Vega-Hernández, Jesús-Ángel Román-Gallego, María-Luisa Pérez-Delgado, Ana-Victoria Torres-García

**Affiliations:** ^1^Department of Statistics, Higher Polytechnic School of Zamora, University of Salamanca, Zamora, Spain; ^2^Department of Computer Science and Automatics, Higher Polytechnic School of Zamora, University of Salamanca, Zamora, Spain; ^3^Department of Personality, Psychological Evaluation and Treatment, Faculty of Psychology, University of Salamanca, Salamanca, Spain

**Keywords:** social services, elderly, multivariate statistics, biplot, STATIS

## Abstract

**Introduction:**

Today’s society is aware that healthy aging favors quality of life in the future, even more so as life expectancy increases in populations such as Europe. As in countries such as Japan, it is necessary for institutions to provide social services to support the elderly, with the aim of achieving an optimal quality of life for these people. The aim of this study is to analyze the different types of social services and activities that certain institutions provide to the elderly in order to find areas for improvement or to propose relationships between them that will benefit both users and institutions.

**Methods:**

Official data from Junta de Castilla y León (Spain) on social services for the elderly in the 9 provinces of the autonomous community of Castilla y León from 2007 to 2021 were analysed using multivariate statistical techniques.

**Results:**

Throughout the period under analysis, there is an association between the number of places in public and private non-profit residential centers for the elderly and the number of places in day-care centers or the number of students in the Inter-University Experience Programme. The variables associated with the telecare programme are related to the number of people under guardianship. On the other hand, three well-differentiated clusters of provinces of Castilla y León were observed.

**Discussion:**

Our findings have implications for the quality of life of the elderly, as the differences in social services in the areas analysed have a direct impact on the health of the elderly.

## 1 Introduction

In the current aging landscape, all countries have been experiencing an increase in the proportion of older people in the population. Life expectancy has increased considerably in recent years, with life expectancy now at or above 60 years for most of the population (World Health Organization [WHO], 2022). Regarding the Spanish data, it is estimated that the population pyramid between 2018 and 2033 will experience a significant decrease in birth and death rates, so it is important to prepare the elderly to stay active, which will allow them to continue contributing to society and, at the same time, benefit from the development of today’s society (Martínez et al., 2021). In the face of this demographic change, Ochoa-Vázquez et al. (2018) point out the importance of preparing society by giving a multidisciplinary approach to old age and aging, being important to consider the different stages of old age and to prepare for active and healthy aging.

This is one of the reasons why the United Nations General Assembly has declared the period 2021–2030 as the Decade for Healthy Aging. It is a global collaborative project led by the World Health Organization (WHO) that aims to unite the efforts of governments, civil society, international agencies, professionals, academia, the media, and the private sector to implement actions, which can achieve the goals of the 2030 Agenda for Sustainable Development and the United Nations Sustainable Development Goals.

The promotion of active and healthy aging is a challenge that we must take up in today’s society. The WHO at the end of the 20th century adopted the term active aging as: the process of optimizing opportunities for health, security and participation, with the aim of improving the quality of life as people age, indicating that it is the process that enhances the potential for physical, mental and social well-being throughout this life cycle. It also promotes the participation of older people in society according to their needs, capabilities and wishes, while providing them with adequate security, protection and care when needed (World Health Organization [WHO], 2002).

To be able to count on more years of life and the restructuring of society not only has implications for the individual person, but also for the community in which you live. Being aware of the socio-demographic changes that are coming, it is possible to plan to make the most out of life (Asamblea Mundial de la Salud, 2016).

Aging is understood as a natural and inevitable process, with old age being a stage of the life cycle influenced by biology but also a social and historical construction, due to the different ways in which the aging process is understood and experienced by different communities (Andrade and Silva, 2016). The aim is not only to live longer, but for people who reach this last stage of life to do so with the highest possible quality of life.

The reality for older people is that there is a high prevalence of unhealthy lifestyles. Sedentary lifestyles are present in 83% of this population group, overweight and obesity in more than 50%, and between 20 and 25% have diabetes (Andrade and Silva, 2016). The prevalence of mental illnesses such as dementia and cognitive impairment is a serious public health problem, being the cause of partial or total alterations in intellectual functions (Arconada et al., 2023). The research carried out by Alonso et al. (2004) on the prevalence of mental disorders in people over 65 years of age indicated that 5.8% had suffered from a mental disorder in the last year. On the other hand, the results of the European study by Andreas et al. (2017) indicate that 47% of older people had experienced a mental disorder in their lifetime, 35.2% in the last year and 23.3% suffered from it at the time the study was carried out. The most frequent mental disorders are anxiety disorders, affective disorders and substance use disorders.

Given this reality, it is necessary to promote active and healthy aging (Palmero Cámara et al., 2014). In this sense, active aging is understood by society as a social right, but it requires compensation strategies to delay the loss of functional capacities, both physical and psychological, which are associated with aging.

As we have already pointed out, some of the problems are sedentary lifestyles, overweight, obesity and psychological problems, so it is necessary to promote healthy habits in the elderly and promote policies to prepare the population for active aging (Martínez et al., 2021). It is necessary to take into account aspects related to physical, psychological and social health so that a better quality of life is possible.

Physical activity is an important aspect, Pinillos (2016) points out that for the development of quality of life in old age, the practice of physical exercise is essential to improve the health of older people. Continuously practicing physical activity improves physical fitness, which has a positive impact on the health and quality of life of older people (Herranz et al., 2013; Medina et al., 2013; Zhao et al., 2013; Enríquez-Reyna et al., 2019).

Relationships are another important aspect, as the older we get, the greater the number of people living alone in their homes. As the years go by, the number of older people living alone, especially women, will increase as a consequence of greater longevity (Pardal et al., 2017; Chen and While, 2019). In Spain, according to data from the Spanish Statistical Office for 2018, there are more than two million people over-65s living alone, which represents almost half of the 4.7 million single-person households. If the marital status of older people is taken into account, the results of the research by Parra-Rizo and Sanchís-Soler (2023) show that widowed and divorced people have a greater/higher frequency of social relationships than married or single people, with widowed people having the most social relationships and single people the least. In terms of satisfaction with social relationships, married people are more satisfied than single, widowed and divorced people, with widowers being more satisfied than single people. These results are in line with those reported by Rondón et al. (2018), which indicate that social relationships, whether due to the frequency of contacts or the size of the social network, are determinant in the health of older people and in the way they manage the illnesses. Recent studies establish a relationship between health status and social relationships (Tsaras et al., 2022).

Taking into account this panorama of the elderly reality, we thought it would be interesting to analyze what is happening in the Autonomous Community of Castilla y León, because according to data from the Spanish Statistical Office [INE] (2022) it is the second autonomous community in Spain with the highest percentage of people over 65 years of age (26.12%), surpassed only by the Principality of Asturias (27.03%). Against this background, it is necessary to analyze the resources available to the autonomous community to meet the needs of this population group.

For the development and support of the elderly, the Community of Castilla y León has a variety of resources which are described in the “Guía para mejorar tu vida” (Guide to improve your life) elaborated by the Consejería de Sanidad y Bienestar Social – Gerencia de Servicios Sociales (Regional Ministry of Health and Social Welfare – Social Services Management) (Junta de Castilla y León, 2003). These resources are related to different areas and adapted to the needs of users, including leisure, culture and participation area, family and community resources, and residential care.

Programmes for non-dependent elderly people include:

**The travel programme for senior citizens of the 60 Club’s Programme for seniors** includes three modalities and a wide offer that allows seniors to travel to 29 destinations.

**The Inter-University Experience Programme**, an initiative of the Junta de Castilla y León in collaboration with all the public and private universities in the region, co-financed by the Management of Social Services, the universities, and the students themselves. It is part of the active ageing and lifelong learning programmes and aims to give older people the possibility of accessing culture and science as a formula for personal growth and social interaction.

**The Integral Programme for Active Ageing** includes activities and services for the prevention of dependency, which are carried out by the public administrations of Castilla y León and private entities financed totally or partially with public funds. It is a unique programme, coordinated and governed by the principles of optimization and rationalization of actions to promote personal autonomy, knowledge of reality and committed participation, among others.

**The Intergenerational Rapprochement Programme** consists of activities in which both older people and young university students participate through agreements among the Social Services Management, the Universities of Burgos, León, Salamanca and Valladolid, and the Town Councils of Burgos, Palencia, Salamanca, Segovia, Soria and Valladolid.

**Thermalism Programme**, whose objective is to promote health care with the knowledge of the cultural and landscape environment of each spa that participates in the programme.

**Programmes and/or aids for dependent and/or disabled people** are aids specifically aimed at people who are in a permanent situation of dependency. This dependency may be due to age, illness or disability, and means that they need constant help from another person in their daily life.

**Dependency Law** offers services and benefits for dependent people and their family environment. After submitting an application to the Social Services of Castilla y León, it is possible to access the following services and benefits:

1. **Services to prevent** dependency situations.
2. **Promotion of personal autonomy.** Rehabilitation and assistance to promote independence.
3. **Home help.** Professional assistance for home cleaning, personal hygiene, etc.
4. **Financial benefit for care in the family environment.** Aid for the carer. This is recognized within the Individual Care Programme.
5. **Financial aid for personal assistance.** For the hiring of a personal assistance service that facilitates the beneficiary’s access to education and work.
6. **Benefit linked to the purchase of a service.** In order to receive the recognized benefit, it is necessary to justify the acquisition of the service.
7. **Financial aid for carer’s rest.** The Junta de Castilla y León covers part of the cost of a dependent person’s stay in a residential center for 1 month a year.
8. **Aid for the elderly.** People over 65 years of age can apply for a series of aid and services, and in some cases being necessary to have a recognized degree of dependency.
9. **Permanent stays in rest homes.** Personalized and uninterrupted care for dependent people with low resources.
10. **Day centers.** Support for family carers. They provide day care and promote the autonomy of the elderly.
11. **“A gusto en casa” (At ease at home) Project.** Initiatives to provide personalized care at home for the elderly in rural areas.
12. **“Viviendas en Red” (Networked Housing) Project.** Municipally-owned housing adapted for the elderly. Residents will pay a symbolic rent and will be able to share a flat if they wish. The aim is to foster a community.

## 2 Materials and methods

### 2.1 Study design

Social services in Spain serve more than four million elderly people (Instituto de Mayores y Servicios Sociales, 2021). Among the most in-demand services are those that allow people to remain in their homes and avoid uprooting from their usual environment. For this reason, a descriptive, exploratory, and longitudinal study was designed to analyze the different types of social services and activities that certain institutions provide to the elderly in order to find areas for improvement that would benefit both users and institutions.

### 2.2 Study setting

In Spain, the number of people aged 65 and over is 9797098, which represents 20.26% of the entire population (data from 1 July 2023) according to the Spanish Statistical Office [INE] (2023). The public social services system is organized as a network to work on coordination, collaboration, and dialogue among all those involved in the process of caring for people.

The management of social services is attributed to the competent bodies of each Autonomous Community and to the Provincial Directorates of the Institute for the Elderly and Social Services, using the resources aimed at the elderly to improve their quality of life through the unified and coordinated development of actions that enable their personal autonomy, guaranteeing the prevention of dependency and care for dependent elderly people. Thus, the Community of Castilla y León has exclusive competence in matters of social assistance, social services, and community development, in accordance with the provisions of Article 70.1.10 of the Statute of Autonomy of Castilla y León, which includes the social rights of the elderly (Ministerio de la Presidencia, Relaciones con las Cortes y Memoria Democrática, 2007).

The setting of this study is the Spanish autonomous community of Castilla y León, one of the largest regions in Europe with 94226 km^2^ and located in the northwestern part of the plateau of the Iberian Peninsula. It is made up of 9 provinces: Ávila, Burgos, León, Palencia, Salamanca, Segovia, Soria, Valladolid, and Zamora, with certain differences among them.

### 2.3 Data

To test the established hypotheses, we selected as target population elderly people (65 and over) living in the autonomous community of Castilla y León (Spain). We worked with the annual information provided by the autonomous community of Castilla y León, which details the official data on social services for the care of the elderly in the 9 provinces of the autonomous community during the last years (2007–2021).

The data has been provided through the web services offered by the Junta de Castilla y León from the portal “Datos abiertos de Castilla y León” (Open Data of Castilla y León). As indicated in its web portal, open data represents a philosophy and practice that aims to make certain data freely available to all people who require it, without restrictions of copyright, patents or other control mechanisms. This data must be published in its original, unprocessed form, well organized and in recognizable formats that facilitate its reuse. In this sense, the public sector generates a wide range of valuable information for citizens, businesses and research groups, ranging from social, economic, geographic, statistical and meteorological data to tourism and business information, as well as data on education. This information has characteristics that make it particularly attractive to the digital content sector, as it is complete, reliable and of high quality. The openness of public sector data allows any person or organization to build on them new ideas that generate additional data, knowledge, process improvements, added value to existing ones or even the creation of new services.

In this sense, we have used and processed the data corresponding to the social services (Junta de Castilla y León, 2022) belonging to the Gerencia de Servicios Sociales - Consejería de Familia e Igualdad de Oportunidades. The data can be downloaded in XLS and CSV format, so that they can be exported in their entirety to different computer tools for subsequent processing. The file with which we have worked offers statistical data on the main variables of the community’s social services that have been delimited to the target age groups of our study, such as those over 65 years of age.

It should be noted that the file contains raw data that had to be filtered to extract the variables necessary for our study, for which different routines were used to select the information we considered relevant and export it to a format that was readable and understandable by the analysis software used.

Table 1 presents the variables analysed in the study together with their assigned codes.

**TABLE 1:** List of codes and variables related to social services for the elderly.

Code	Social services variables
Total Cost Telecare	Total cost of the telecare programme (in euros).
Usu Telecare	Number of telecare service users.
Annual Pensions Amount	Annual amount of old age and sickness pensions (in euros).
Students Experience	Number of students at the Inter-University Experience Programme.
Associations CyL	Number of associations of elderly people in Castilla y León.
PartTerm60Club	Number of participants in thermalism trips of the 60’s Club.
Part60Club	Number of participants in 60’s Club trips.
Benef	Number of beneficiaries of old age and sickness benefits.
Seniors Supervised	Number of elderly people under guardianship.
DayCareCentres	Number of places in day-care centres for elderly people.
ResPrivProfit	Number of places in residential centres for the elderly (Private for profit).
ResPrivNon-Profit	Number of places in residential care homes for the elderly (Private non-profit).
ResPublic	Number of places in residential centres for the elderly (Public).
Members60Club	Number of members of the 60’s Club.

In addition, official data on life expectancy at birth from the European Statistical Office (Eurostat)^1^ and the Spanish Statistical Office (INE)^2^ were used to show the situation of older people. According to the United Nations, life expectancy is defined as “the number of years a newborn can expect to live if the age-specific mortality patterns prevailing at the time of birth remained the same throughout life.”

And, to find out about the possible benefits that currently exist for the elderly in the region under study, data from the Ministry of Social Rights and Agenda 2030^3^ were analyzed.

### 2.4 Analysis

First, the aging trend in Castilla y León (Spain) was analysed to determine the exact situation of the elderly in the region. For this purpose, a descriptive analysis was carried out. Official data on the elderly population, life expectancy and basic services for the elderly were studied, both at the European level and in the Spanish region to be analysed. Subsequently, the evolution over time of social services for the elderly in Castilla y León was analysed. This was carried out by means of frequency distribution tables and line diagrams, representing on the X axis the period and on the Y axis the quantitative value of the variable corresponding to each social service.

To analyze the common structure of social services for the elderly from 2007 to 2021, the STATIS method (Escoufier, 1973; Des Plantes, 1976) was applied. It is a suitable exploratory technique for three-way data analysis, in this case, information on social services for the elderly from 2007 to 2021 for the 9 provinces of the region studied. This method comprises three stages: the interstructure, the analysis of the compromise, and the intrastructure. For this paper, we will focus only on the first two.

The first stage is the study of interstructure. It is the analysis of the configuration of k points that correspond to the k matrices in a graphical representation of one or more flat Euclidean images of projection of k points for the study of the relation between different matrices, in our case representing different years, where similarity (positive correlations are visualized by small acute angles between variables) indicate that the variables of the social services maintain a stable behavior with respect to the provinces of the region during the study period. A matrix is built of scalar products between tables (the vector covariance matrix), where the element in row k and column l is $\text{Cov}=\left({\text{X}}_{\text{k}},{\text{X}}_{\text{l}}\right)=\mathrm{tr}\,\left({\text{X}}_{\text{k}}^{\text{t}}\,{\text{D}}_{\text{n}}\,{\text{X}}_{\text{l}}\,{\text{D}}_{\text{p}}\right)$, where X_k_ is the kth table of the sequence, and D_p_ and D_n_ are the two metrics for the columns and rows, respectively. This way, we can determine if the matrices have similar structures.

The second stage is the analysis of the compromise. It is the closest matrix to configurations of k matrices, where a linear transformation of each data table is performed so that each matrix becomes a column vector, stacking their variables one on top of another. This matrix combines the consensus structure of the k data tables representing the common structure of the variables in these tables. So, through the application of a factorial analysis with the principal components method to this matrix, we can plot the structure to interpret the representation of the averages for the variables.

To study the relationships between social services for the elderly in the provinces of Castilla y León each year, an HJ-biplot analysis was applied (Galindo, 1986). Biplots (Gabriel, 1971) are statistical exploratory multidimensional techniques that represent the joint structure of the individuals (provinces) and variables (social services) of a multivariate data matrix X. We applied HJ-biplot because offers a representation in a low dimensional space of a matrix X_nxp_. Let X = UDV^t^ be the usual singular value decomposition (SVD) of X with U and V orthogonal matrices and D = diag(λ_1_,…,λ_*p*_) containing the singular values. Let J and H be the matrices of the first two columns of UD and VD, respectively. This method allows, by the suitable selection of markers, j_i_ = (j_i_,…,j_n_) for its rows and h_j_ = (h_j_,…,h_n_) for its columns, to represent simultaneously in the same Euclidean space the rows and columns with the highest quality of representation. For its interpretation, we have to keep several guidelines going, so row markers (provinces) are represented as points and column markers (social services variables) as vectors. In this way, we can visualize a set of provinces with similar behaviors, interpreting the distance between points as similarity, so that provinces closer to other provinces present similar profiles. To describe the relationships between social services for the elderly, acute angles between vectors are associated with a high positive correlation. And, to classify the provinces in reference to social services, we can order the different province in relation to each variable with the orthogonal projections of the points (provinces) on the vectors (social services variables). Remember that the interpretation is subject to the quality of representation of the elements in the observed subspace.

Finally, to analyze the social services for the elderly in the different provinces, the normality of the data was tested using the Shapiro-Wilk test. All the variables did not have a normal distribution, except for participants in thermalism trips of the 60’s Club (see auxiliary table). So, to compare the quantitative variables of the social services for the elderly, the Kruskal-Wallis test was applied. And to identify differences between provinces, a posteriori pairwise comparisons were performed.

Statistical analyses were performed, using Excel (version 2308), IBM SPSS Statistics (version 28.0), R-Studio (version 4.1.2) and MultBiplot software (Vicente-Villardón et al., 2016).

## 3 Results

### 3.1 Situation of older people

In recent years, life expectancy in Europe has been increasing, with a value of 79.8 years in 2010, 80.8 years in 2014, and 81.3 years in 2019, although in 2020 there was a decrease with a life expectancy value of 80.4 years (Eurostat, 2023). For the year 2048, life expectancy is estimated at 82.3 years (World Health Organization [WHO], 2023). In Castilla y León, the same trend in life expectancy has also been observed over the last few years, as shown in Table 2. This is due to demographic ageing as a result of the evolution of the birth rate and the decrease in mortality (Gerencia de Servicios Sociales, 2017).

**TABLE 2:** Life expectancy at birth by province in recent years, separated by sex.

	Ávila	Burgos	León	Palencia	Salamanca	Segovia	Soria	Valladolid	Zamora	Castilla y León
**Both**										
2007	81.07	82.09	81.18	80.96	82.60	82.47	82.42	81.93	82.49	81.87
2008	82.07	82.09	81.78	82.22	82.79	82.85	82.79	81.88	82.54	82.22
2009	82.96	82.34	82.57	81.32	82.96	83.12	83.36	82.66	82.97	82.67
2010	82.23	83.17	82.58	81.93	83.68	83.35	83.85	82.47	83.45	82.90
2011	82.72	82.96	82.97	82.29	83.94	83.40	84.00	83.04	83.38	83.17
2012	83.31	82.89	82.91	82.39	83.77	83.65	83.65	83.16	83.22	83.16
2013	82.88	83.57	82.73	83.18	84.32	83.76	84.47	83.69	83.27	83.48
2014	83.14	83.60	83.13	82.17	84.58	84.10	84.04	83.87	83.40	83.61
2015	82.56	84.22	83.27	82.98	84.58	83.29	84.07	83.24	83.60	83.58
2016	83.27	84.09	83.50	83.12	84.48	84.02	83.05	84.19	83.22	83.82
2017	83.30	83.81	83.70	83.13	84.42	83.94	84.82	83.97	83.05	83.82
2018	83.42	84.06	83.58	82.81	84.69	83.95	84.55	84.29	83.13	83.92
2019	83.93	84.50	83.63	83.25	84.70	84.70	84.52	84.63	83.40	84.20
2020	82.21	83.15	82.50	81.11	82.54	81.50	82.31	82.94	82.88	82.53
2021	83.53	84.52	83.51	82.41	84.74	84.06	83.69	83.97	84.11	83.93
**Males**										
2007	78.54	78.95	77.75	77.27	79.52	79.75	80.21	78.92	79.75	78.82
2008	79.76	78.74	78.74	78.95	79.93	80.17	80.18	78.85	79.64	79.26
2009	80.38	79.15	79.23	77.85	80.02	80.81	80.28	80.06	80.15	79.70
2010	79.19	80.17	79.20	78.34	80.81	80.81	81.41	79.45	80.40	79.86
2011	80.66	80.18	79.90	79.21	80.98	80.82	81.09	79.93	80.43	80.29
2012	80.73	79.60	79.79	79.16	81.09	81.36	80.74	80.21	80.72	80.25
2013	80.01	80.60	79.90	80.28	81.70	81.16	82.35	81.05	80.45	80.74
2014	80.61	80.45	80.30	79.18	81.76	81.30	81.11	81.23	80.94	80.83
2015	79.76	81.22	80.78	80.00	82.23	80.22	81.34	80.39	80.67	80.83
2016	81.26	81.15	80.41	79.95	81.87	81.33	80.82	81.44	80.47	81.03
2017	81.29	81.09	81.02	79.93	81.86	81.54	81.82	81.33	80.49	81.18
2018	81.07	81.36	80.79	80.22	82.18	81.45	82.06	81.94	80.30	81.33
2019	81.13	81.63	80.91	80.02	82.05	82.36	82.32	82.18	80.75	81.53
2020	79.79	80.29	79.74	78.16	79.89	78.36	80.11	80.19	80.30	79.78
2021	80.70	81.59	80.61	79.34	81.67	81.44	81.46	81.06	81.43	81.07
**Females**										
2007	83.80	85.45	84.72	84.86	85.65	85.32	84.70	84.88	85.32	85.02
2008	84.50	85.73	84.84	85.57	85.63	85.57	85.62	84.84	85.57	85.25
2009	85.75	85.79	85.98	85.00	85.90	85.44	86.89	85.17	85.95	85.72
2010	85.59	86.36	86.06	85.82	86.52	85.96	86.46	85.45	86.71	86.04
2011	84.91	85.87	86.07	85.50	86.87	86.14	87.14	86.08	86.49	86.10
2012	86.12	86.47	86.06	85.88	86.40	86.00	86.85	86.03	85.82	86.15
2013	86.07	86.69	85.56	86.16	86.85	86.44	86.68	86.21	86.30	86.26
2014	85.94	87.02	85.95	85.31	87.39	87.06	87.29	86.37	85.99	86.44
2015	85.65	87.38	85.73	86.16	86.86	86.62	87.10	86.02	86.75	86.39
2016	85.43	87.17	86.64	86.60	87.03	86.83	85.51	86.85	86.16	86.66
2017	85.47	86.69	86.36	86.49	86.92	86.49	88.08	86.52	85.80	86.51
2018	85.96	86.90	86.38	85.46	87.12	86.53	87.29	86.50	86.23	86.54
2019	87.03	87.59	86.34	86.64	87.34	87.07	87.01	86.96	86.27	86.93
2020	84.90	86.33	85.36	84.47	85.28	85.06	84.86	85.68	85.72	85.45
2021	86.73	87.66	86.47	85.77	87.88	86.88	86.18	86.86	87.02	86.92

Spanish Statistical Office (2021).

According to the Social Services portal, in a few years Spain will have 19.7 million people over the age of 64 and the number of centenarians will be almost double what it is today.

Among the basic services aimed at the elderly are: information and guidance, home help, living unit, alternative accommodation or prevention and insertion. The most recent data on the elderly (2021) show a majority use (61.9%) of the information and guidance service, followed by the home help service (17.8%) (Ministerio de Derechos Sociales y Agenda 2030, 2021).

### 3.2 Telecare for the elderly

**FIGURE 1:**
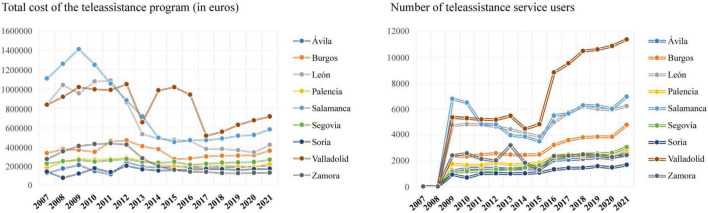
Evolution of costs and number of users of the telecare programme in Castilla y León from 2007 to 2021.

Figure 1 presents the evolution since 2007 of the costs and the number of users of the telecare programme in the region under study by province.

At the beginning of the period analysed there was an increasing trend in the total costs (in euros) of the telecare programme in Castilla y León. In the province of Salamanca, a maximum of 1411092 euros was observed in 2009, but thereafter there was a sharp drop until 2014 when it started to stabilize. However, the province of León had a relative minimum in 2009 (954412 euros) which managed to increase the following 2 years, although from 2011 to 2013 it suffered a steep decline. The average cost of the telecare programme in Valladolid was 833049 euros during the time period studied, with relative peaks above one million euros; however, in 2013 and 2017 there were very serious decreases. The provinces of Ávila, Burgos, Palencia, Segovia, Soria, and Zamora show more stable trends, with few variations and costs that in no case exceed 470000 euros over these years. Since 2017 in all the provinces of Castilla y León, there has been a slight increase in these costs or at least a stabilization, with the exception of León which had a small decrease until 2020 but then showed an increase of 77208 euros the following year.

The number of people using the telecare service in Castilla y León has also varied over the period analysed (see Figure 1). From 2009 onward, there was a slight decrease in the number of users of this service in all provinces, with the exception of Zamora, which was maintained and in 2013 stands out with a maximum of 3191 people. In León, Salamanca, Valladolid and Zamora, the number of users clearly decreased until 2015. Subsequently, as in the rest of the provinces, there was a gradual increase in the number of users, except in Valladolid and Salamanca, which had large increases (3984 and 1981 more people the following year, respectively).

### 3.3 Old age and illness

**FIGURE 2:**
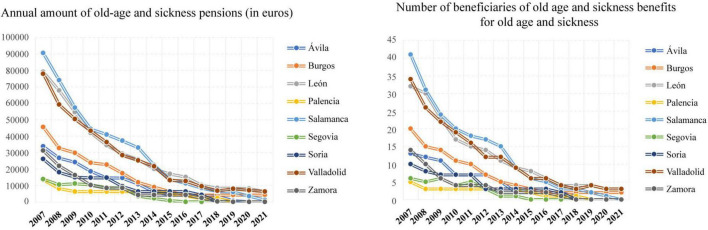
Evolution of pensions and beneficiaries for old age and illness in Castilla y León from 2007 to 2021.

Figure 2 shows how since 2007 the annual amount of old age and sickness pensions, in euros, has decreased in all the provinces analysed. This decline was very pronounced in 2008. In 2015 and 2018 there was also a slight drop and since then it has been maintained. The same trend can be observed in the number of people benefiting from old age and sickness benefits.

The number of older people under guardianship since 2007 has been increasing, although in a specific year the amount decreases (for example, the year 2020), it is observed that the following year it increases again (see Table 3). The largest number of people under guardianship is observed in the province of Valladolid, followed by Salamanca and Burgos with more than 1030 people.

**TABLE 3:** Distribution of elderly people under guardianship in Castilla y León from 2007 to 2021.

Year	Ávila	Burgos	León	Palencia	Salamanca	Segovia	Soria	Valladolid	Zamora	Total
2007	12	43	21	26	10	22	22	101	16	273
2008	8	45	26	30	11	28	20	97	14	279
2009	17	45	30	33	16	43	31	91	15	321
2010	21	45	36	34	24	46	26	85	23	340
2011	19	49	29	27	32	46	38	74	18	332
2012	27	53	29	34	50	41	38	103	31	406
2013	30	68	48	46	58	41	47	133	55	526
2014	39	80	52	46	81	44	68	109	54	573
2015	33	60	58	52	81	41	52	129	53	559
2016	27	77	68	50	89	38	53	136	49	587
2017	32	77	70	51	87	42	41	132	46	578
2018	45	84	72	49	99	39	49	158	53	648
2019	56	106	83	52	134	33	48	171	62	745
2020	66	94	79	53	109	24	46	182	58	711
2021	73	106	93	57	157	30	54	183	69	822
Total	505	1032	794	640	1038	558	633	1884	616	7700

### 3.4 60’s club

**FIGURE 3:**
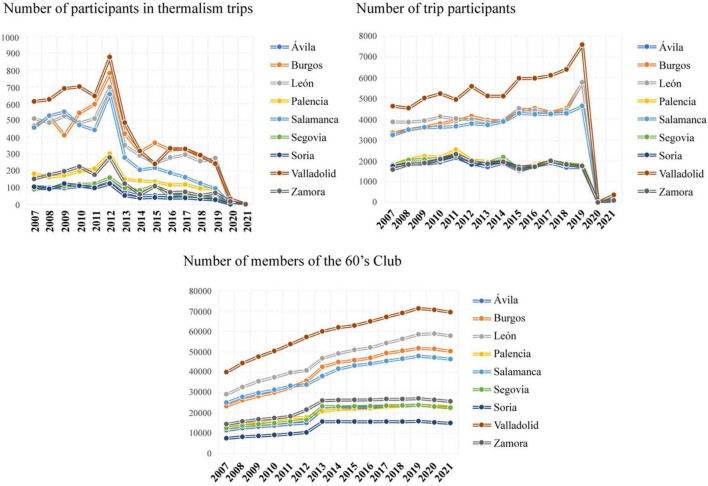
Evolution of participation in the Castilla y León 60’s Club from 2007 to 2021.

The trend of seniors choosing to become members of the 60’s Club is increasing, as shown in the graph on the right of Figure 3. Regarding participation in spa trips, although there was an increase in 2012, it has declined and continues to decline (graph on the left of Figure 3). However, the participation of those over 60 years of age has undergone slight ups and downs with an increasing trend, taking into account that from 2019 to 2020 there were no trips due to the COVID-19 pandemic (graph in the middle of Figure 3).

### 3.5 Residential centers for the elderly

**FIGURE 4:**
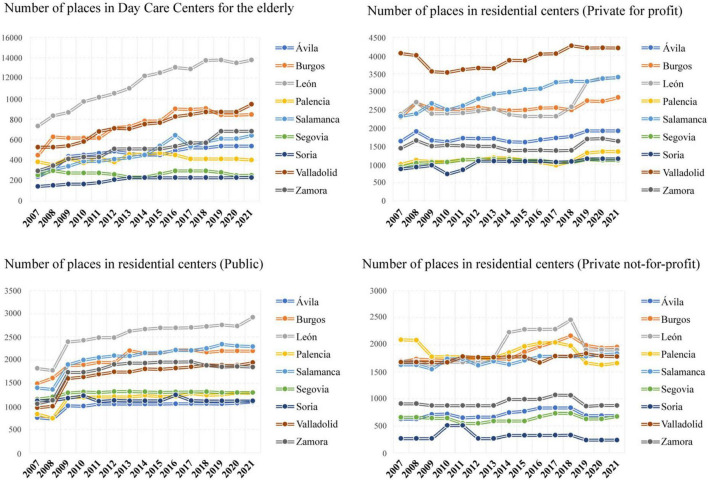
Evolution of vacancies in residential centers for the elderly in Castilla y León from 2007 to 2021.

Over the last few years, the number of places in residential centers for the elderly has remained fairly constant. Public places suffered a drop in 2008 but rebounded in the following year and have remained stable since then. A slight increase was only observed in 2013 in the province of Burgos, in 2016 in Soria and recently in León. Moreover, the highest number of public places is observed in León, followed by Salamanca and Burgos, while the lowest number is observed in Segovia, Palencia, Soria, and Ávila. Private places, both for-profit and not-profit, also remained stable throughout the last years, but suffered some variations between the years 2009–2012 and 2018–2019. The province with the highest number of private for-profit places during the period studied was Valladolid and the one with the lowest number of non-profit places was Soria. Regarding the number of Day Care Centers for the Elderly, a growing trend has been observed, more pronounced in León, Burgos, or Valladolid than in the rest of the provinces (see Figure 4).

### 3.6 Associations and programmes for the elderly

**FIGURE 5:**
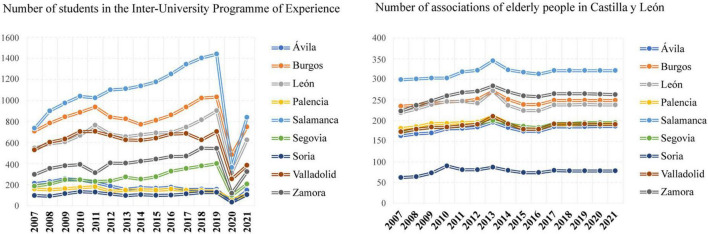
Evolution of the number of students in the Inter-University Programme of Experience and associations for the elderly in Castilla y León from 2007 to 2021.

Figure 5 shows in the evolution in recent years of the number of the Inter-University Programme of Experience students, as well as the associations for older people in Castilla y León. It is interesting to note the increasing trend in the number of students in the Inter-University Programme of Experience and the clear decrease in 2019 and 2020 in all provinces. In 2011, the slight increase in Burgos and León and the decrease in Salamanca and Zamora are noteworthy. Throughout the period studied, the number of associations of elderly people in Castilla y León has been constant, only slightly increased in 2013. The province with the largest number of associations is Salamanca and the one with the fewest is Soria.

### 3.7 Common structure of social services for the elderly from 2007 to 2021: STATIS method

**FIGURE 6:**
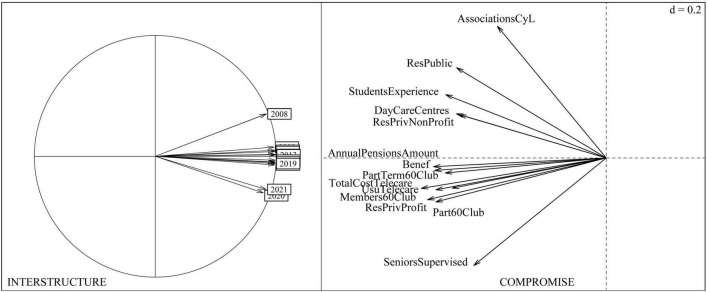
Interstructure and compromise of social services for the elderly from 2007 to 2021.

The interstructure analysis (Figure 6) indicates that the data matrices corresponding to the period of time studied have similar structures. The year 2008 presents a larger acute angle with the other years, suggesting instability or certain differences in social services for the elderly in that year with respect to the rest. This also occurs with the periods 2020 and 2021.

The compromise presents strong relationships among the various social services for the elderly in Castilla y León. Figure 6 shows a strong relationship between the number of public and private non-profit places in residential centers for the elderly, the number of places in Day Care Centers for the elderly and the number of students in the Inter-University Programme of Experience over the period studied (2007–2021), and in turn, with the number of associations for the elderly in Castilla y León. The rest of the social services: the annual amount of old age and sickness pensions (in euros), the number of people benefiting from old age and sickness benefits, the number of participants in thermalism trips of the 60’s Club, the total cost of the telecare programme (in euros), the number of users of the telecare service, the number of members of the 60’s Club, the total cost of the telecare programme (in euros), the number of users of the telecare service, the number of members of the 60’s Club, and the number of users of the telecare service, the number of members of the 60’s Club, the number of participants in 60’s Club trips and the number of places in residential centers for the elderly (private for profit) are highly correlated over the period 2007–2021, showing a weaker association with the number of elderly people under guardianship.

### 3.8 Social services for the elderly each year: HJ-biplot

In order to have an overall view of the relationships of the variables on social services for the elderly in the 9 provinces of Castilla y León, an HJ-biplot analysis was applied to the data sets for each year.

For a correct application of this technique, it is necessary to show the explained variance values and the relative contribution of the factor to the element, which will indicate the position of the axes and their subsequent interpretation; both are shown in Tables 4, 5.

**TABLE 4:** Eigenvalues and explained variance in each year.

Axis		Eigenvalue	% Var	% Accum		Eigenvalue	% Var	% Accum		Eigenvalue	% Var	% Accum
1	2 0 0 7	71.27	68.53	68.53	2 0 0 8	70.87	68.14	68.14	2 0 0 9	79.68	71.14	71.14
2		14.21	13.66	82.19		13.20	12.69	80.83		13.64	12.18	83.32
3		7.63	7.34	89.52		7.35	7.07	87.90		8.29	7.40	90.72
4		6.11	5.88	95.40		6.67	6.41	94.31		4.89	4.36	95.08
5		2.52	2.43	97.83		2.87	2.76	970.72		2.30	2.05	97.13
6		1.42	1.37	99.20		1.97	1.90	98.97		1.86	1.66	98.79
7		0.76	0.73	99.92		1.01	0.97	99.94		1.04	0.93	99.72
8		0.08	0.08	100.00		0.07	0.06	100.00		0.31	0.28	100.00
1	2 0 1 0	81.00	72.32	72.32	2 0 1 1	82.38	73.55	73.55	2 0 1 2	82.98	74.09	74.09
2		13.28	11.86	84.18		12.93	11.55	85.10		12.39	11.06	85.15
3		6.47	5.78	89.96		5.88	5.25	90.35		6.61	5.90	91.05
4		4.68	4.18	94.14		4.57	4.08	94.42		4.79	4.28	95.33
5		2.61	2.33	96.47		2.44	2.18	96.60		2.47	2.20	97.53
6		1.99	1.77	98.24		1.90	1.70	98.30		1.59	1.42	98.95
7		1.68	1.50	99.74		1.67	1.49	99.79		1.13	1.01	99.96
8		0.30	0.27	100.00		0.24	0.22	100.00		0.05	0.04	100.00
1	2 0 1 3	82.40	73.57	73.57	2 0 1 4	82.87	73.99	73.99	2 0 1 5	79.47	70.95	70.95
2		11.40	10.18	83.75		12.63	11.27	85.27		14.13	12.61	83.57
3		7.78	6.94	90.69		7.51	6.71	91.97		7.86	7.02	90.59
4		4.19	3.74	94.43		3.68	3.28	95.26		4.96	4.43	95.02
5		2.98	2.66	97.10		2.40	2.14	97.39		2.51	2.24	97.26
6		2.18	1.95	99.04		1.70	1.52	98.92		1.73	1.54	98.80
7		0.87	0.77	99.82		0.74	0.66	99.57		0.92	0.82	99.62
8		0.21	0.18	100.00		0.48	0.43	100.00		0.43	0.38	100.00
1	2 0 1 6	80.47	71.85	71.85	2 0 1 7	82.19	73.39	73.39	2 0 1 8	83.92	74.92	74.92
2		14.01	12.51	84.36		12.43	11.10	84.48		12.58	11.24	86.16
3		7.05	6.30	90.66		7.66	6.84	91.32		7.68	6.86	93.02
4		5.45	4.87	95.52		4.80	4.28	95.61		2.97	2.65	95.67
5		2.44	2.18	97.70		2.17	1.94	97.54		2.23	2.00	97.67
6		1.45	1.30	98.99		1.38	1.24	98.78		1.49	1.33	99.00
7		0.84	0.75	99.74		0.89	0.80	99.58		0.78	0.70	99.70
8		0.29	0.26	100.00		0.47	0.42	100.00		0.34	0.31	100.00
1	2 0 1 9	86.77	77.47	77.47	2 0 2 0	73.51	70.69	70.69	2 0 2 1	75.98	73.05	73.05
2		11.15	9.95	87.43		15.12	14.54	85.22		13.34	12.83	85.88
3		7.78	6.95	94.37		6.44	6.19	91.42		8.72	8.39	94.27
4		2.80	2.50	96.88		4.27	4.11	95.53		2.22	2.13	96.40
5		1.69	1.51	98.38		1.77	1.70	97.23		1.68	1.62	98.02
6		1.03	0.92	99.30		1.61	1.55	98.77		1.01	0.97	98.99
7		0.48	0.43	99.73		0.92	0.88	99.65		0.75	0.73	99.72
8		0.30	0.27	100.00		0.36	0.35	100.00		0.29	0.28	100.00

**TABLE 5:** Relative contribution of the factor to the element in each year.

	2007		2008		2009		2010		2011	
Variable	Axis 1	Axis 2	Axis 1	Axis 2	Axis 1	Axis 2	Axis 1	Axis 2	Axis 1	Axis 2
Total Cost Telecare	804	42	801	54	807	30	818	32	879	19
Usu Telecare	81	776	577	130	853	20	853	32	917	2
Annual Pensions Amount	843	21	810	41	867	4	850	0	833	2
Students Experience	799	81	807	72	775	60	795	56	830	25
Associations CyL	375	343	404	295	420	398	366	449	336	486
PartTerm60Club	969	12	957	10	956	27	909	42	903	23
Part60Club	918	51	928	52	883	109	891	100	916	70
Benef	844	25	822	35	863	0	852	1	810	5
SeniorsSupervised	266	685	255	693	147	785	252	648	222	677
DayCareCentres	573	6	552	2	433	3	504	12	545	28
ResPrivProfit	792	146	800	108	872	54	875	45	867	36
ResPrivNon-Profit	476	1	474	8	565	0	608	5	646	8
ResPublic	334	292	352	192	622	129	643	163	678	191
Members60Club	914	72	896	88	895	87	910	75	916	46
	**2012**		**2013**		**2014**		**2015**		**2016**	
**Variable**	**Axis 1**	**Axis 2**	**Axis 1**	**Axis 2**	**Axis 1**	**Axis 2**	**Axis 1**	**Axis 2**	**Axis 1**	**Axis 2**
Total Cost Telecare	918	7	913	1	765	172	639	313	741	215
Usu Telecare	936	0	852	9	950	0	911	54	838	95
Annual Pensions Amount	802	8	768	6	842	11	767	4	696	21
Students Experience	788	64	746	105	704	56	684	110	636	151
Associations CyL	363	418	420	437	295	396	273	424	246	521
PartTerm60Club	920	12	861	40	848	0	659	83	843	3
Part60Club	891	89	900	66	933	40	934	27	942	13
Benef	780	33	758	1	854	6	752	3	717	53
Seniors Supervised	363	553	456	446	508	327	581	293	765	124
Day Care Centres	529	30	517	21	553	129	650	70	670	97
ResPrivProfit	898	56	912	37	873	73	839	62	863	42
ResPrivNon-Profit	633	3	675	15	649	118	637	78	527	168
ResPublic	654	210	625	187	647	239	663	233	616	244
Members60Club	897	66	900	54	939	10	945	12	959	3
Total Cost Telecare	854	36	814	83	765	88	724	119	787	48
Usu Telecare	839	82	815	128	802	124	813	156	833	88
Annual Pensions Amount	750	54	845	1	942	7	892	1	820	44
Students Experience	626	155	577	161	632	195	646	184	584	281
Associations CyL	279	554	307	417	299	540	273	314	268	589
PartTerm60Club	814	1	807	4	764	0	219	513	377	417
Part60Club	936	34	913	60	931	36	29	6	703	223
Benef	712	103	881	6	918	27	878	1	714	72
Seniors Supervised	805	73	709	214	774	55	731	189	747	19
Day Care Centres	630	45	670	72	657	46	670	80	705	30
ResPrivProfit	853	39	853	82	938	20	949	11	952	4
ResPrivNon-Profit	574	136	638	124	730	40	719	89	703	79
ResPublic	639	241	697	210	723	199	689	230	695	184
Members60Club	963	3	963	13	972	14	985	3	987	8

**FIGURE 7:**
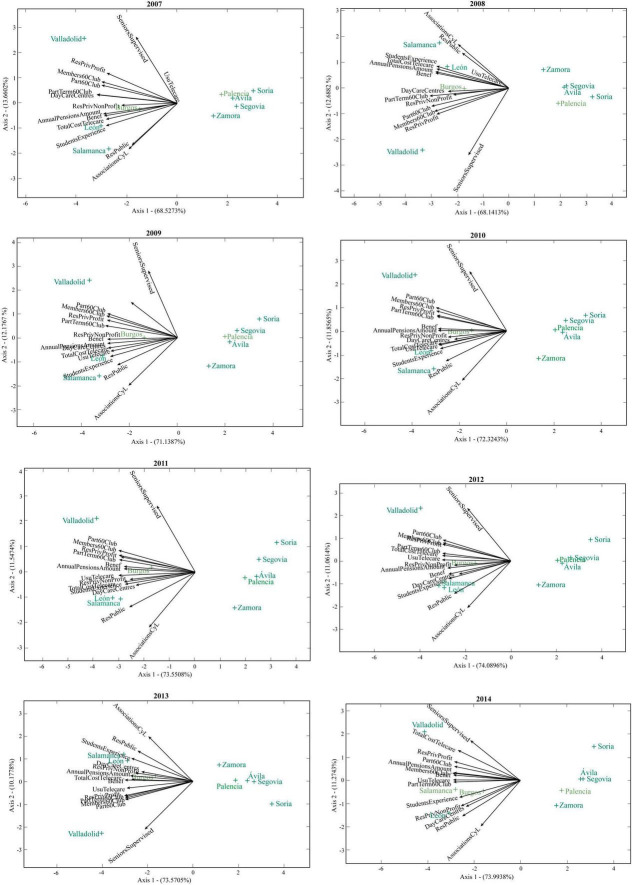
HJ-biplots of the social services for the elderly of Castilla y León in each year (2007–2014).

**FIGURE 8:**
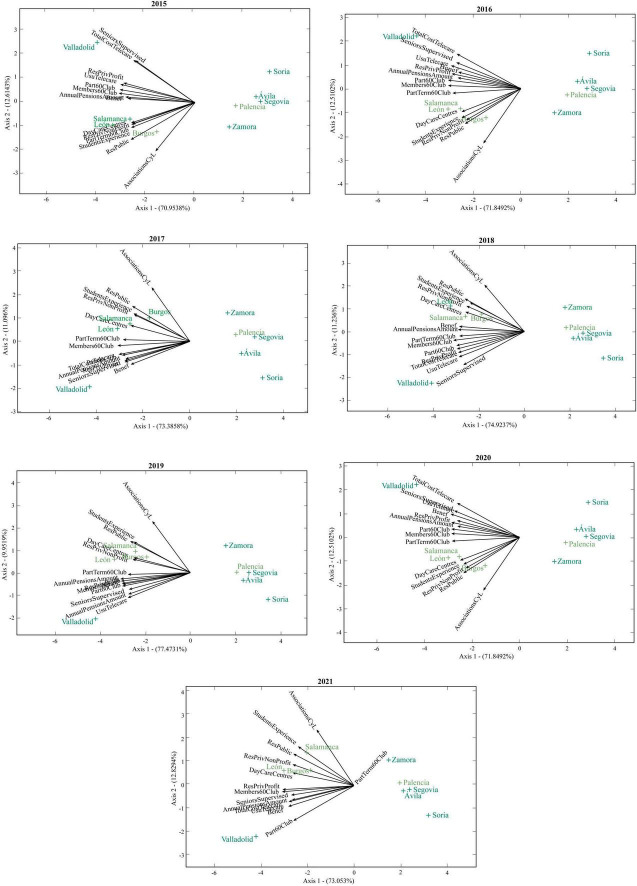
HJ-biplots of the social services for the elderly of Castilla y León in each year (2015–2021).

In Figure 7 and Figure 8, the points of each biplot correspond to the provinces of Castilla y León and the vectors refer to the variables associated with social services for the elderly. Thus, those vectors that form acute angles correspond to positive correlations and the points with a similar position in the plane correspond to similar values in the different vectors. Provinces close to or in orthogonal projection to the end of the vector will indicate high scores in that social service.

In the first 2 years (2007 and 2008), the number of associations of elderly people in Castilla y León was closely related to the number of public places in residential centers for the elderly. However, this association decreased slightly in the following years and in 2019 they were closely related to the number of students at the Inter-University Programme of Experience. These services are independent of the number of elderly people under guardianship in Castilla y León, which in 2015 and 2016 were found to be highly related to the costs of the telecare service for elderly people.

Although the rest of variables on social services are closely related to each other, two groups of variables with very strong correlations can be observed up to 2010. The first is formed by the number of places in residential centers for the elderly (private for profit), the number of members of the 60’s Club, the number of participants in thermalism trips of the 60’s Club and the number of participants in trips of the 60’s Club. The second is formed by the annual amount of pensions for old age and illness, the number of beneficiaries of old age and illness benefits, the total cost of the telecare programme and the number of students at the Inter-university Programme of Experience. In 2011, 2012 and 2013 the variables of both groups are highly correlated with each other, and no groups are distinguished. However, in 2014 and 2015 the first group of variables is observed to be more differentiated.

As of 2016, the number of places in Day Care Centers for the Elderly, the number of students in the Inter-University Programme of Experience and the number of places in residential centers for the elderly (private non-profit and public) are highly correlated and, in turn, related to the number of associations for the elderly in Castilla y León. In 2021, the independence between the number of associations of elderly people in Castilla y León and the number of participants in trips of the 60’s Club stands out.

The provinces of Valladolid, León and Salamanca show high values for social services for the elderly. In contrast, Zamora, Ávila, Palencia, Segovia and Soria show very low values that indicate scarce social resources in these provinces. The best values in this group correspond to the first 3 provinces, while the worst value corresponds to Soria.

The elderly in the provinces of Salamanca and León have more associations, many public places in residential centers and have participated a lot in the Inter-University Programme of Experience.

Valladolid is the province with more people under guardianship and from 2015 it has had very high costs in telecare, unlike provinces such as Zamora or Segovia. Social services for the elderly in Valladolid have been very high over time, with the number of members of the 60’s Club standing out in 2021.

In addition, taking into account the time period analysed (from 2007 to 2021), statistically significant differences (*p* < 0.001) are also observed among the number of users of the telecare service, the annual amount of old age and sickness pensions (in euros), the number of participants in 60’s Club trips (including thermal trips), the number of people benefiting from old age and sickness benefits, and the number of elderly people under guardianship.

The main differences in the telecare service are observed between the years 2007 and 2008 with the rest of the years analysed, highlighting greater inequalities from 2016 onward (*p* < 0.001). Differences in participation in the 60’s Club trips are observed in the last years (*p* < 0.001), although in terms of thermal trips they also extend to 2019 and highlights 2012 with the period from 2014 to 2019 (*p* < 0.05).

### 3.9 Social services for the elderly in different provinces

There are statistically significant differences (*p* < 0.001) among the provinces of Castilla y León in social services for the elderly.

Regarding the money invested in the telecare programme, the differences among León, Salamanca and Valladolid and Ávila, Zamora and Segovia stand out (*p* < 0.001). Significant differences were also observed between Burgos and Ávila (*p* < 0.001), Zamora (*p* < 0.014), Palencia (*p* < 0.023), Salamanca (*p* < 0.019) and Valladolid (*p* < 0.009); Soria was clearly different from the other provinces, except for Ávila (*p* < 0.378). Similarly, in the number of users of the telecare service, there are highly significant differences between León, Salamanca and Valladolid and Soria, Ávila, Segovia, Palencia, and Zamora (*p* < 0.001 for the first three and p < 0.01 for the last two). There are differences of Burgos with Soria (*p* = 0.001), Ávila (*p* = 0.027) and Valladolid (*p* = 0.027), and of Soria with Zamora (*p* = 0.029).

Concerning the annual amount of old age and sickness pensions, there were significant differences between Salamanca, Valladolid and León with Segovia (*p* < 0.001), Palencia (*p* ≤ 0.001), Zamora (*p* < 0.01), Soria (*p* < 0.05) and Ávila (*p* < 0.05). In addition, differences were observed between Burgos and Segovia (*p* = 0.018) and Palencia (*p* = 0.029). On the other hand, there were significant differences in the number of students in the Inter-University Experience Programme between Valladolid, León, Burgos and Salamanca and Soria, Palencia and Ávila (*p* < 0.001). Differences are observed between Soria and Segovia (*p* = 0.005), Zamora with Soria (*p* < 0.001), Palencia (*p* = 0.005), Ávila (*p* = 0.036), Burgos (*p* = 0.001) and Salamanca (*p* = 0.001), Segovia with Valladolid and León (*p* < 0.011 and *p* = 0.003 respectively), and Valladolid with Salamanca (*p* = 0.018).

In terms of the number of associations of elderly people in Castilla y León, there were significant differences between León, Burgos, Zamora and Salamanca with Soria (*p* < 0.001), Ávila (*p* < 0.001), Valladolid (*p* < 0.01), Segovia (*p* < 0.01) and Palencia (*p* < 0.02). Also of Soria with Valladolid, Segovia and Palencia (p = 0.011, p = 0.003 and p = 0.001 respectively) and of Salamanca with León and Burgos (*p* = 0.006 and *p* = 0.036 respectively). The provinces of León, Burgos and Valladolid show significant differences in the number of participants in thermalism trips of the 60’s Club with Soria (*p* < 0.001), Ávila (*p* < 0.001), Segovia (*p* ≤ 0.001), Zamora (*p* < 0.01) and Palencia (*p* < 0.05). Significant differences were observed between Salamanca and Soria (*p* < 0.001), Ávila (*p* = 0.001), Segovia (*p* = 0.003) and Zamora (*p* = 0.041), and between Soria and Segovia (*p* = 0.046). Regarding the number of participants in 60 Club trips, the significant differences between Salamanca, Burgos, León and Valladolid with Ávila (*p* ≤ 0.001), Soria (*p* ≤ 0.002), Zamora (*p* ≤ 0.003), Segovia (*p* < 0.01) and Palencia (*p* < 0.02) stand out.

Regarding the number of people benefiting from old age and sickness benefits, there are significant differences in the provinces of Salamanca, Valladolid and León with Segovia (*p* < 0.001), Palencia (*p* ≤ 0.001), Zamora (*p* < 0.01), Soria (*p* < 0.02) and Ávila (*p* < 0.05). Differences are also observed in Burgos with Segovia and Palencia (*p* = 0.017 and *p* = 0.026 respectively). There are clear significant differences between Burgos and Valladolid with Ávila (*p* < 0.001), Segovia (*p* ≤ 0.001), Soria (*p* = 0.013 and *p* < 0.001), Zamora (*p* = 0.014 and *p* < 0.001) and Palencia (*p* = 0.021 and *p* < 0.001) in the number of older people under guardianship. There are also differences between Salamanca with Ávila, Segovia and Valladolid (*p* = 0.005, *p* = 0.014 and *p* = 0.002), Ávila with León (*p* = 0.036), Valladolid with León and Burgos (*p* < 0.001 and *p* = 0.019). With respect to the number of day care places for the elderly, significant differences were observed between Valladolid, Burgos and León with the rest of the provinces of Castilla y León (*p* < 0.01), as well as between Ávila, Salamanca and Zamora with Soria and Segovia (*p* < 0.01).

In the number of places in private residential centers for the elderly, there were significant differences between Burgos, Salamanca and Valladolid with Soria (*p* < 0.001), Segovia (*p* < 0.001), Palencia (*p* < 0.001), Zamora (*p* < 0.01) and Ávila (*p* < 0.05) between Zamora, Ávila and León with Soria (*p* < 0.01) and Segovia (*p* < 0.05), Palencia with Ávila and León (*p* < 0.01), León with Valladolid (*p* = 0.014) and Burgos with Valladolid (*p* = 0.023). In the number of places in private non-profit residential centers for the elderly, Salamanca, Valladolid, Burgos, Palencia and León differ significantly from Soria (*p* < 0.001), Segovia (*p* < 0.001), Ávila (*p* < 0.001) and Zamora (*p* < 0.01), and between Soria and Zamora (*p* = 0.002). And in the number of places in public residential centers for the elderly, Burgos, Salamanca and León differed significantly from Ávila (*p* < 0.001), Soria (*p* < 0.001), Palencia (*p* < 0.001), Segovia (*p* < 0.001), and León (*p* < 0.001). 001), Segovia (*p* ≤ 0.001) and Valladolid (*p* < 0.01), in addition, Valladolid and Zamora differ statistically from Ávila (p < 0.01), Soria (*p* < 0.01) and Palencia (*p* < 0.05), and Zamora from León (*p* = 0.003).

Finally, in the number of members of the 60’s Club there are significant differences between Salamanca, Burgos, León and Valladolid with the rest of the provinces of Castilla y León (*p* < 0.001, except with Zamora which *p* < 0.01), there are also differences between Soria and Zamora (*p* = 0.005) and Salamanca and Valladolid (*p* < 0.05).

## 4 Discussion

This study analyzes the different types of social services for the elderly in one of the main regions of Spain, the autonomous community of Castilla y León, in order to find areas for improvement. To our knowledge, it is the first to apply the STATIS method to have a global and common vision over the years.

The analysis of social services for the elderly from 2007 to the present shows some instability in 2008, which may be due to the economic crisis suffered at that time. Variations were also observed in the last years (2020 and 2021), which may be due to the post-pandemic recovery. This study indicates the existence of a strong association between the number of public and private non-profit places in residential centers for the elderly, the number of places in Day Care Centers for the elderly and the number of students in the Inter-University Programme of Experience throughout the period studied, and in turn, with the number of associations for the elderly in Castilla y León. The rest of the social services are strongly related throughout the period, showing a weaker association with the number of older people under guardianship. And significant inequalities were observed in the social services for the elderly among the provinces studied.

Regarding the telecare programme for the elderly, Art. 15 of Law 39/2006, of December 14, on the Promotion of Personal Autonomy and Care for Dependent Persons, establishes the social services for the promotion of personal autonomy and care for dependency, among which the telecare service is contemplated, with the aim of providing assistance to the beneficiaries by means of communication and information technologies, with the support of the necessary personal means, in immediate response to emergency situations, or situations of insecurity, loneliness and isolation (BOE, 2006). The evolution of this programme in Castilla y León, in general terms, has been increasing since 2017 or has stabilized. The telecare service provided by the Spanish Red Cross in Castilla y León, as Fermoso et al. (2011) point out, is a fairly efficient resource because of how its organizational structure is designed and the available human resources that manage to minimize the total costs of the service per user, by using the Queuing Theory methodology.

The Spanish population pyramid is experiencing a significant decrease in birth rate and mortality since 2018 and the forecasts are that it will follow this same line until 2033, this is going to see an increase in the number of people over 65 years of age and, therefore, it is going to increase diseases and mental disorders in this population group. On the other hand, the number of pensions due to this cause will also increase, the data analysed with respect to these variables reflect a decrease in the annual amount since 2007, in all the provinces of the Community of Castilla y León. In the coming years, the trend is that the provinces of Valladolid, Salamanca and Burgos will need more resources to cover the demand.

Although the number of senior citizens who are members of the 60’s Club has fluctuated slightly over the years, the trend is growing, the offer of these activities and the destinations are very varied both nationally and internationally. Taking part in activities that facilitate social relations is an important aspect of active aging, favoring a better promotion of the health of this group.

Intergenerational programmes encourage participation in the community, promote healthier aging in all generations involved in the programmes (Butts and Chana, 2007). Older people benefit from these programmes, they exercise more, facilitating the burning of calories, they are more active, they suffer fewer falls, they are more independent and dependence on other people is delayed. In relation to young people, they learn from the experiences of older people, they know and share their life habits, sharing time and space that facilitates the improvement and quality of life and social relations (Maccallum et al., 2006; Pinazo and Kaplan, 2007).

Older people who participate in intercollegiate programmes of experience, benefit from active aging and lifelong learning, it is a possibility to access culture and science that enhances personal growth and social interaction. Alsubaie et al. (2019), point out that social support acts positively in the face of stressful life situations and promotes mental health, it plays an essential role in the quality of life, as it collaborates with the feeling of being appreciated and connected to the environment. It has a high impact on people with depression and it can be concluded that both its presence and absence are determinant in mental health issues (Zabala, 2021). Continuing to promote these programmes in the Community of Castilla y León is important and necessary for the elderly population. Knowles et al. (2001) point out that “the need to know influences motivation to learn, learning outcomes and subsequent motivation to take advantage of learning.”

As a consequence of a longer-lived and aging population in Castilla y León, it is necessary not to neglect health care, especially primary care and other services for the elderly, which are increasingly in demand, largely due to the difficulties of assistance and care by the intergenerational family support network. This has resulted in a greater increase in the number of people living alone in this autonomous community. Modino and Fanjul (2020) point out that this population group, as they get older, are a more vulnerable population, so they increasingly need community care services provided at home, which would delay the need to leave the home to enter nursing homes. Resources such as home help, to assist in the performance of household chores and other basic activities in the daily life of the elderly, such as personal hygiene, companionship or telecare to facilitate communication in the event of an emergency, proximity services (meals at home or laundry service, pharmacy, food supply or financial services), especially necessary for the elderly in rural areas, as well as day centers, which facilitate social interaction and relationships with other people of generation. In addition to facilitating other necessary services such as sociocultural animation, physiotherapy, occupational therapy or cognitive stimulation, among others, it would delay or avoid admission to nursing homes.

We conclude that providing quality social services for the elderly is of great importance. Our study shows clear associations between some of the services, which could be used to implement better action strategies. However, we have observed large differences in social services for the elderly among the provinces studied. We observed two well differentiated groups: one formed by Valladolid, Salamanca, León and Burgos, with more resources in terms of social services for the elderly. And another group formed by Zamora, Palencia, Ávila, Segovia and Soria, with much more limited resources. Therefore, appropriate strategies should be proposed to reduce these great inequalities between the provinces of the same region.

This study has several strengths, but also limitations. One advantage is the use of multivariate statistical methods that provide a global view of the formation of various data sets in a simple way. In addition, another strength is the study of social services from 2007 to the present, allowing the analysis of the evolution of the various services for the elderly. We have also worked with official data from the reference agencies. Although a limitation of the research is that it does not make any projections, it is only a descriptive and exploratory study. In the future, the study could be extended to other regions.

## Data availability statement

Publicly available datasets were analyzed in this study. This data can be found here: https://datosabiertos.jcyl.es/web/jcyl/set/es/sociedad-bienestar/servicios_sociales/1284230077683 https://ec.europa.eu/eurostat https://www.ine.es/index.htm.

## Author contribution

M-CV-H: Conceptualization, Formal analysis, Visualization, Writing – original draft, Writing – review and editing. J- R-G: Conceptualization, Data curation, Resources, Writing – review and editing. M-LP-D: Conceptualization, Supervision, Writing – review and editing. A-VT-G: Conceptualization, Writing – original draft, Writing – review and editing.

## Footnotes

1
https://ec.europa.eu/eurostat

2
https://www.ine.es/index.htm

3
https://www.mdsocialesa2030.gob.es/index.htm

## References

[B1] Alonso J. Angermeyer M. C. Bernert S. Bruffaerts R. Brugha T. S. Bryson H. (2004). Prevalence of mental disorders in Europe: Results from the European study of the epidemiology of mental disorders (ESEMeD) project. *Acta Psychiatr. Scand.* Suppl. 420 21–27. 10.1111/j.1600-0047.2004.00327.x 15128384

[B2] Alsubaie M. M. Stain H. J. Webster L. A. D. Wadman R. (2019). The role of sources of social support on depression and quality of life for university students. *Int. J. Adolesc. Youth* 24:13. 10.1080/02673843.2019.1568887

[B3] Andrade C. I. Silva K. (2016). Avaliação da qualidade de vida de idosos atendidos em um ambulatório de Geriatria da região nordeste do Brasil, São Paulo. *Rev. Bras. Clin. Med*. 11 129–134.

[B4] Andreas S. Schulz H. Volkert J. Dehoust M. Sehner S. Suling A. (2017). Prevalence of mental disorders in elderly people: The European MentDis_ICF65+ study. *Br. J. Psychiatry* 210 125–131. 27609811 10.1192/bjp.bp.115.180463

[B5] Arconada L. R. Tobías C. C. Luengo A. P. Romea N. B. Ibáñez L. G. Sánchez A. M. B. (2023). Revisión sistemática. Evidencia de prevalencia de demencia y deterioro cognitivo en personas mayores de 65 años en hogares de ancianos. *Rev. Sanitaria Invest.* 4:5

[B6] Asamblea Mundial de la Salud, 69 (2016). *Acción Multisectorial Para Un Envejecimiento Saludable Basado En El Ciclo De Vida: Proyecto De Estrategia Y Plan De Acción Mundiales Sobre El Envejecimiento Y La Salud: Informe De La Secretaría.* Geneva: Organización Mundial de la Salud.

[B7] BOE (2006). Ley 39/2006, De 14 De Diciembre, De Promoción De La Autonomía Personal Y Atención A Las Personas En Situación De Dependencia. Available online at: https://www.boe.es/eli/es/l/2006/12/14/39 (accessed May 17, 2023).

[B8] Butts D. M. B. A. Chana K. B. S. W. (2007). Intergenerational programs promote active aging. *J. Active Aging* 34, 34–39.

[B9] Chen Y. While A. E. (2019). Older people living alone in Shanghai: A questionnaire survey of their life experience. *Health Soc. Care Commun*. 27 260–269. 10.1111/hsc.12648 30160058

[B10] Des Plantes L. H. (1976). *Structuration Des Tableaux à Trois Indices De La Statistique: Théorie et Application d’une Méthode d’analyse Conjointe*. France: University of Montpellier.

[B11] Enríquez-Reyna M. C. Carranza D. Navarro R. (2019). Nivel de actividad física, masa y fuerza muscular de mujeres mayores de la comunidad: Diferencias por grupo etario. *Retos* 35 121–125.

[B12] Escoufier Y. (1973). Le traitement des variables vectorielles. *Biometrics* 29 751–760. 10.2307/2529140

[B13] Eurostat (2023). *Dataset Life Expectancy by Age and Sex.* Available online at: https://ec.europa.eu/eurostat/databrowser/view/demo_mlexpec/default/table?lang=en [accessed September 26, 2023].

[B14] Fermoso F. J. P. García J. M. G. González A. G. (2011). Aplicaciones de la Teoría de Colas a la provisión óptima de servicios sociales: El caso del servicio de Teleasistencia. *Stud. Appl. Econ.* 29:26.

[B15] Gabriel K. R. (1971). The biplot graphic display of matrices with application to principal component analysis. *Biometrika* 58 453–467.

[B16] Galindo M. P. (1986). Una alternativa de representación simultanea: HJ-Biplot. *Qüestiió* 10, 13–23.

[B17] Gerencia de Servicios Sociales (2017). *Estrategia De Prevención de la Dependencia Para Personas Mayores y Promoción del Envejecimiento Activo 2017-2021*. de Castilla y León: Consejería de Familia e Igualdad de Oportunidades.

[B18] Herranz I. Lirio J. Portal E. Arias E. (2013). La actividad física como elemento de participación y calidad de vida en las personas mayores. *Escritos de Psicol.* 6 13–19. 10.5231/psy.writ.2013.1906

[B19] Instituto de Mayores y Servicios Sociales (2021). *Los Servicios Sociales Dirigidos a Las Personas Mayores en España: Datos a 31 de diciembre de 2021.* Madrid: Ministerio de Derechos Sociales y Agenda 2030.

[B20] Junta de Castilla y León (2003). *Memoria 2003. Gerencia de servicios sociales*. Available online at: https://serviciossociales.jcyl.es/web/jcyl/binarios/509/911/Parte_I__Servicios_sociales.pdf

[B21] Junta de Castilla y León (2022). *Estadísticas de servicios sociales de Castilla y León.* Castilla y León: Gerencia de Servicios Sociales - Consejería de Familia e Igualdad de Oportunidades.

[B22] Knowles M. S. Holton E. F. Swanson R. A. (2001). *Andragogía: el Aprendizaje de los Adultos.* México: Oxford University Press.

[B23] Maccallum J. Palmer D. Wright P. Cumming-Potvin W. Northcote J. Booker M. Tero C. (2006). *Community Building Through Intergenerational Exchange Programs.* Canberra, NSW: National youth Affairs Research Scheme

[B24] Martínez N. Santaella E. Rodríguez-García A.-M. (2021). Beneficios de la actividad física para la promoción de un envejecimiento activo en personas mayores. Revisión bibliográfica (Benefits of physical activity for the promotion of active aging in elderly. Bibliographic review). *Retos* 39 829–834. 10.47197/retos.v0i39.74537

[B25] Medina C. Janssen I. Campos I. Barquera S. (2013). Physical inactivity prevalence and trends among Mexican adults: Results from the National Health and Nutrition Survey (ENSANUT) 2006 and 2012. *BMC Public Health* 13:1063. 10.1186/1471-2458-13-1063. 24215173 PMC3883516

[B26] Ministerio de Derechos Sociales y Agenda 2030 (2021). *Memoria 2021. Previsiones 2022. Plan concertado para el desarrollo de prestaciones básicas de servicios sociales.* Madrid: Ministerio de Derechos Sociales y Agenda 2030

[B27] Ministerio de la Presidencia, Relaciones con las Cortes y Memoria Democrática (2007). *Estatuto de Autonomía de Castilla y León.* Castilla y León: BOE

[B28] Modino J. M. D. Fanjul A. P. (2020). Despoblación, envejecimiento y políticas sociales en Castilla y León. *Rev Galega Econ.* 29 1–18.

[B29] Ochoa-Vázquez J. Cruz-Ortiz M. Pérez-Rodríguez M. Cuevas-Guerrero C. E. (2018). El envejecimiento: Una mirada a la transición demográfica y sus implicaciones para el cuidado de la salud. *Rev. Enferm. IMSS* 26 273–280.

[B30] Palmero Cámara M. D. C. Gañán Adánez Á. Luis Rico M. I. Torre Cruz T. D. L. Baños Martínez V. Escolar Llamazares M. D. C. (2014). Influencia de las actividades intergeneracionales universitarias en la calidad de vida de jóvenes y mayores. *Int. J. Dev. Educ. Psychol.* 1:158.

[B31] Pardal L. P. i Montells L. P. Álvarez L. R. (2017). Mayores que viven solos y malnutrición. Estudio SOLGER. *Atención Primaria* 49 450–458. 10.1016/j.aprim.2016.10.007 28153387 PMC6875975

[B32] Parra-Rizo M. A. Sanchís-Soler G. (2023). Impacto del nivel de actividad física y estado civil en la vulnerabilidad de la salud psicofísica en personas mayores de 60 años: Mayor atención sociosanitaria. *Cuadernos Psicol Del Deporte* 23(2), 273–291.

[B33] Pinazo S. Kaplan M. (2007): “Los beneficios de los programas intergeneracionales,” in *Programas Intergeneracionales. Hacia una Sociedad Para Todas Las Edades: 70- 101*, ed. Sánchez M (Barcelona: Fundación la Caixa).

[B34] Pinillos M. (2016). Efectos positivos del entrenamiento de karate en las capacidades cognitivas asociadas a la edad. *Rev. Int. Med. y Ciencias de la Actividad Física y el Deporte* 16 537–559. 10.15366/rimcafd2016.63.009

[B35] Rondón L. M. Arizala A. García F. J. (2018). El significado de las relaciones sociales como mecanismo para mejorar la salud y calidad de vida de las personas mayores, desde una perspectiva interdisciplinar. *Rev. Española de Geriatría y Gerontol.* 53 268–273. 10.1016/j.regg.2018.01.005 29703555

[B36] Spanish Statistical Office (2021). *Life expectancy at birth by province, by sex*. Castilla y León: INE. Available online at: https://www.ine.es/en/

[B37] Spanish Statistical Office [INE] (2022). *Demography and population*. Spain: INE. Available online at: https://www.ine.es/en/

[B38] Spanish Statistical Office [INE] (2023). *Demography and population*. Spain: INE. Available online at: https://www.ine.es/en/

[B39] Tsaras K. Tsiantoula M. Papagiannis D. Papathanasiou I. V. Chatzi M. Kelesi M. (2022). The effect of depressive and insomnia symptoms in quality of life among community-dwelling older adults. *Int. J. Environ. Res. Public Health* 19:13704. 10.3390/ijerph192013704 36294280 PMC9603389

[B40] Vicente-Villardón (2016). *MULTBIPLOT: A package for Multivariate Analysis using Biplots. Departamento de Estadística.* Salamanca: Universidad de Salamanca

[B41] World Health Organization (2002). *The world health report 2002: reducing risks, promoting healthy life*. World Health Organization.

[B42] World Health Organization [WHO] (2022). *World health statistics 2022*. Geneva: Departmental News.

[B43] World Health Organization [WHO] (2023). *World Health Statistics 2023: Monitoring Health for the SDGs, Sustainable Development Goals*. Geneva: World Health Organization

[B44] Zabala A. G. G. (2021). Apoyo social y envejecimiento activo: Aproximaciones desde la perspectiva de la psicología positiva. *Rev. Científica Arbitrada de la Fundación MenteClara* 6:239

[B45] Zhao X. Wang Z. Zhang J. Hua J. He W. Zhu S. (2013). Estimation of total body skeletal muscle mass in Chinese adults: Prediction model by Dual-Energy X Ray Absorptiometry. *PLoS One* 8:e53561. 10.1371/journal.pone.0053561 23308254 PMC3538629

