# Supplementary material for: Social services for the elderly: a multivariate perspective study
Source: Front Psychol. 2023 Nov 24;14:1297349. doi: 10.3389/fpsyg.2023.1297349 (PMC10704906; doi:10.3389/fpsyg.2023.1297349)
Supplement: Supplementary file 1 [file Table_1.docx]

**Auxiliary table.** Normality tests using the Shapiro-Wilk test.

| Variable |  | value | p |  | Variable |  | value | p |
| --- | --- | --- | --- | --- | --- | --- | --- | --- |
| TotalCostTelecare | Ávila | 0.896 | 0.081 |  | Benef | Ávila | 0.868 | 0.031 |
|  | Burgos | 0.933 | 0.307 |  |  | Burgos | 0.813 | 0.006 |
|  | León | 0.823 | 0.007 |  |  | León | 0.858 | 0.022 |
|  | Palencia | 0.897 | 0.087 |  |  | Palencia | 0.868 | 0.031 |
|  | Salamanca | 0.829 | 0.009 |  |  | Salamanca | 0.894 | 0.078 |
|  | Segovia | 0.975 | 0.923 |  |  | Segovia | 0.774 | 0.002 |
|  | Soria | 0.861 | 0.025 |  |  | Soria | 0.881 | 0.049 |
|  | Valladolid | 0.874 | 0.039 |  |  | Valladolid | 0.867 | 0.031 |
|  | Zamora | 0.817 | 0.006 |  |  | Zamora | 0.797 | 0.003 |
| UsuTelecare | Ávila | 0.871 | 0.035 |  | SeniorsSupervised | Ávila | 0.930 | 0.277 |
|  | Burgos | 0.873 | 0.037 |  |  | Burgos | 0.899 | 0.092 |
|  | León | 0.753 | 0.001 |  |  | León | 0.926 | 0.241 |
|  | Palencia | 0.815 | 0.006 |  |  | Palencia | 0.877 | 0.043 |
|  | Salamanca | 0.826 | 0.008 |  |  | Salamanca | 0.946 | 0.459 |
|  | Segovia | 0.930 | 0.269 |  |  | Segovia | 0.884 | 0.054 |
|  | Soria | 0.873 | 0.037 |  |  | Soria | 0.960 | 0.698 |
|  | Valladolid | 0.889 | 0.066 |  |  | Valladolid | 0.935 | 0.325 |
|  | Zamora | 0.781 | 0.002 |  |  | Zamora | 0.876 | 0.041 |
| AnnualPensionsAmount | Ávila | 0.886 | 0.059 |  | DayCareCentres | Ávila | 0.791 | 0.003 |
|  | Burgos | 0.822 | 0.007 |  |  | Burgos | 0.913 | 0.151 |
|  | León | 0.859 | 0.023 |  |  | León | 0.902 | 0.102 |
|  | Palencia | 0.895 | 0.078 |  |  | Palencia | 0.950 | 0.520 |
|  | Salamanca | 0.889 | 0.064 |  |  | Salamanca | 0.922 | 0.206 |
|  | Segovia | 0.803 | 0.004 |  |  | Segovia | 0.922 | 0.204 |
|  | Soria | 0.901 | 0.098 |  |  | Soria | 0.735 | 0.001 |
|  | Valladolid | 0.868 | 0.032 |  |  | Valladolid | 0.918 | 0.182 |
|  | Zamora | 0.808 | 0.005 |  |  | Zamora | 0.940 | 0.388 |
| StudentsExperience | Ávila | 0.885 | 0.057 |  | ResPrivProfit | Ávila | 0.841 | 0.013 |
|  | Burgos | 0.927 | 0.247 |  |  | Burgos | 0.907 | 0.123 |
|  | León | 0.918 | 0.180 |  |  | León | 0.712 | <0.001 |
|  | Palencia | 0.796 | 0.003 |  |  | Palencia | 0.898 | 0.090 |
|  | Salamanca | 0.943 | 0.422 |  |  | Salamanca | 0.930 | 0.270 |
|  | Segovia | 0.956 | 0.629 |  |  | Segovia | 0.820 | 0.007 |
|  | Soria | 0.808 | 0.005 |  |  | Soria | 0.844 | 0.014 |
|  | Valladolid | 0.739 | 0.001 |  |  | Valladolid | 0.900 | 0.094 |
|  | Zamora | 0.932 | 0.288 |  |  | Zamora | 0.874 | 0.038 |
| g.l. = 15 |  |  |  |  |  |  |  |  |

**Auxiliary table** **- continued.** Normality tests using the Shapiro-Wilk test.

| Variable |  | value | p |  | Variable |  | value | p |
| --- | --- | --- | --- | --- | --- | --- | --- | --- |
| AssociationsCyL | Ávila | 0.933 | 0.304 |  | ResPrivNonProfit | Ávila | 0.894 | 0.078 |
|  | Burgos | 0.843 | 0.014 |  |  | Burgos | 0.871 | 0.034 |
|  | León | 0.866 | 0.029 |  |  | León | 0.844 | 0.014 |
|  | Palencia | 0.869 | 0.033 |  |  | Palencia | 0.904 | 0.108 |
|  | Salamanca | 0.864 | 0.028 |  |  | Salamanca | 0.931 | 0.286 |
|  | Segovia | 0.936 | 0.331 |  |  | Segovia | 0.946 | 0.465 |
|  | Soria | 0.920 | 0.190 |  |  | Soria | 0.743 | 0.001 |
|  | Valladolid | 0.887 | 0.060 |  |  | Valladolid | 0.814 | 0.006 |
|  | Zamora | 0.873 | 0.037 |  |  | Zamora | 0.773 | 0.002 |
| PartTerm60Club | Ávila | 0.948 | 0.494 |  | ResPublic | Ávila | 0.580 | <0.001 |
|  | Burgos | 0.955 | 0.604 |  |  | Burgos | 0.793 | 0.003 |
|  | León | 0.929 | 0.267 |  |  | León | 0.795 | 0.003 |
|  | Palencia | 0.951 | 0.548 |  |  | Palencia | 0.616 | <0.001 |
|  | Salamanca | 0.935 | 0.321 |  |  | Salamanca | 0.776 | 0.002 |
|  | Segovia | 0.952 | 0.564 |  |  | Segovia | 0.609 | <0.001 |
|  | Soria | 0.895 | 0.080 |  |  | Soria | 0.629 | <0.001 |
|  | Valladolid | 0.950 | 0.522 |  |  | Valladolid | 0.710 | <0.001 |
|  | Zamora | 0.960 | 0.701 |  |  | Zamora | 0.650 | <0.001 |
| Part60Club | Ávila | 0.632 | <0.001 |  | Members60Club | Ávila | 0.767 | 0.001 |
|  | Burgos | 0.759 | 0.001 |  |  | Burgos | 0.874 | 0.039 |
|  | León | 0.678 | <0.001 |  |  | León | 0.923 | 0.213 |
|  | Palencia | 0.692 | <0.001 |  |  | Palencia | 0.866 | 0.029 |
|  | Salamanca | 0.668 | <0.001 |  |  | Salamanca | 0.895 | 0.079 |
|  | Segovia | 0.668 | <0.001 |  |  | Segovia | 0.762 | 0.001 |
|  | Soria | 0.630 | <0.001 |  |  | Soria | 0.743 | 0.001 |
|  | Valladolid | 0.775 | 0.002 |  |  | Valladolid | 0.925 | 0.227 |
|  | Zamora | 0.685 | <0.001 |  |  | Zamora | 0.774 | 0.002 |
| g.l. = 15 |  |  |  |  |  |  |  |  |
